# The Role of Surfactin Production by *Bacillus velezensis* on Colonization, Biofilm Formation on Tomato Root and Leaf Surfaces and Subsequent Protection (ISR) against *Botrytis cinerea*

**DOI:** 10.3390/microorganisms9112251

**Published:** 2021-10-28

**Authors:** Alexandra Stoll, Ricardo Salvatierra-Martínez, Máximo González, Michael Araya

**Affiliations:** 1 Laboratorio de Microbiología Aplicada, Centro de Estudios Avanzados de Zonas Áridas (CEAZA), La Serena 1720256, Chile; maximo.gonzalez@ceaza.cl; 2 Programa de Doctorado en Biología y Ecología Aplicada, Departamento de Biología, Universidad de La Serena, La Serena 1720170, Chile; rsmesteban@gmail.com; 3 Centro de Investigación y Desarrollo Tecnológico en Algas CIDTA, Universidad Católica del Norte, Coquimbo 1781421, Chile; mmaraya@ucn.cl

**Keywords:** colonization pattern, population density, surfactin production, ISR

## Abstract

Many aspects regarding the role of lipopeptides (LPs) in bacterial interaction with plants are not clear yet. Of particular interest is the LP family of surfactin, immunogenic molecules involved in induced systemic resistance (ISR) and the bacterial colonization of plant surfaces. We hypothesize that the concentration of surfactin produced by a strain correlates directly with its ability to colonize and persist on different plant surfaces, which conditions its capacity to trigger ISR. We used two *Bacillus velezensis strains* (BBC023 and BBC047), whose antagonistic potential in vitro is practically identical, but not on plant surfaces. The surfactin production of BBC047 is 1/3 higher than that of BBC023. Population density and SEM images revealed stable biofilms of BBC047 on leaves and roots, activating ISR on both plant surfaces. Despite its lower surfactin production, strain BBC023 assembled stable biofilms on roots and activated ISR. However, on leaves only isolated, unstructured populations were observed, which could not activate ISR. Thus, the ability of a strain to effectively colonize a plant surface is not only determined through its production of surfactin. Multiple aspects, such as environmental stressors or compensation mechanisms may influence the process. Finally, the importance of surfactin lies in its impacts on biofilm formation and stable colonization, which finally enables its activity as an elicitor of ISR.

## 1. Introduction

Currently, several strains of *Bacillus* sp. are used as biocontrol agents against crop disease [1,2,3,4]. These strains exhibit different biocontrol mechanisms [5], of which antibiosis and the induction of plant resistance (ISR) are considered the most important. Some of these mechanisms of action are directly linked to the synthesis of lipopeptides (LPs) by *Bacillus* spp. [1,6,7,8]. LPs are secondary metabolites formed by cyclic peptides linked to a lipid tail or other lipophilic molecules, which self-assemble in diverse configurations and therefore have high structural diversity. Three families, namely iturins, fengycins and surfactins, have been distinguished and characterized to have different capacities, e.g., as antibiotics (bacillomycin and fengycin) or elicitors of ISR [9,10].

In the context of biocontrol, it has been postulated that both direct antagonism and ISR require efficient colonization of plant surfaces [7,11,12]. The colonization of plants is a complex process that involves the attraction to and establishment of microorganisms in different plant compartments. The attraction phase, particularly in the roots, occurs through chemotaxis induced by plant exudates, while establishment involves swarming motility and biofilm production [13,14,15,16]. For both mechanisms, a dependence on the production of the immunogenic LP surfactin has been suggested [2,6,17].

According to previous studies, surfactin contributes to both direct and indirect antagonism and is key to plant colonization [2,18,19]. Zeriouh et al. [6] reported that although the LP antibiotics bacillomycin and fengycin, synthesized by *B. velezensis* UMAF6614, generate biocontrol of powdery mildew by direct action, mutations that affect the production of surfactin in this strain decrease its biocontrol capacity despite it continuing to produce the antibiotic LPs. This synergistic effect between the different lipopeptide families was also described for other *Bacillus* spp. strains [2,19], enhancing the dual functionality of surfactin during antibiosis and colonization. Several authors reported a physiological threshold dose of surfactin (10 µm/mL), required to trigger ISR in plants [7,20]. For the colonization of and biofilm formation on plant surfaces, such a threshold has not been published. Recently, Therien et al. [21] suggested that in *B. subtilis* strains biofilm formation could be independent from surfactin production.

In a previous study, we compared the biocontrol effect generated by two strains of *B. velezensis* (strains BBC023 and BBC047) against *Botrytis cinerea* (a necrotrophic fungus of great global importance) in vitro and in vivo [22]. We observed that under in vitro conditions, the antagonistic potential of both strains was practically identical, while the results between the strains differed when they were required to establish on plant surfaces.

Thus, we postulate that these differences could be correlated with the concentration of surfactin produced by a strain, which could determine the colonization ability of the respective strain and its permanence on different plant surfaces. Thereby, our work evaluated the production of surfactin and the colonization in time of two *B. velezensis* strains (BBC023 and BBC047) in a pathosystem comprising a tomato (host) and *B. cinerea*.

## 2. Materials and Methods

### 2.1. Bacterial Strains, Broth and Growth Conditions

The native strains of *B. velezensis* BBC023 and BBBC047 [20], the model strain FZB42 (producer of surfactin A, bacillomycin D and fengycin A and B; [23]) and its CH1 mutant deficient in the production of surfactin [24] were used. All bacterial strains were grown in Luria–Bertani (LB) broth for inoculum production, and 1.5% agar was used for solid plates. For the growth of the CH1 mutant, 1 μg/mL erythromycin was added to the growth medium.

For plant inoculation, bacterial strains were grown in a MOLP medium [25] at 30 °C and 170 rpm for 48 h (~10^9^ colony-forming units (CFU) per mL). Finally, the bacterial concentration was adjusted to ~10^7^ CFU/mL.

### 2.2. Quantification of Surfactin Production with HPLC

The LPs were obtained by the acid precipitation method [26]. The different strains were grown in flasks with 100 mL of MOLP medium for 48 h at 30 °C and 170 rpm. Subsequently, the media were centrifuged at 10,000 rpm for 15 min, and the supernatant was recovered for the next steps. LPs were precipitated by adding 3 N HCl to the supernatant until pH 2.0 was reached. Afterwards, the samples were incubated for 30 min at 4 °C, and the LPs were precipitated by centrifugation at 10,000 rpm for 15 min. Finally, the supernatant was discarded, and the LPs were resuspended in 1 mL of methanol (100%). This fraction was injected into a Jasco CO-2065 Plus HPLC system (SpectraLab Scientific Inc., Markham, ON, Canada) equipped with a 5 μm particle size Sunniest C18 250 × 4.6 mm column (CromaNik Technologies Inc., Osaka, Japan) and a UV detector. Surfactin concentrations were determined by calibration curves according to the method of Ali et al. [27]. Quantification was performed in triplicate for each strain using the same protocol.

### 2.3. Biofilm Formation, Motility and Adherence

Biofilm formation was evaluated on Petri dishes with solid MSgg medium (2.5 mM PBS, pH 7, 100 mM MOPS, pH 7, 50 μM FeCl_3_, 2 mM MgCl_2_, 50 μM MnCl_2_, 1 μM ZnCl_2_, 2 μM thiamine, 50 mg phenylalanine, 0.5% glycerol, 0.5% glutamate and 700 μM CaCl_2_ [28]). Each inoculated Petri dish was subsequently incubated at 37 °C for 48 h [22]. Motility was evaluated using semisolid LB medium (0.75% agar), while adherence was characterized in 15 × 150 mm tubes with 10 mL of MOLP medium, which were incubated for 48 h at 30 °C [29].

The initial inoculum for each assay was 3 μL of bacterial culture grown in liquid LB for 12 h, adjusted to 10^7^ CFU/mL.

### 2.4. Characterization of Surfactin Profiles with UHPLC-MS

To identify the LP isoforms, 20 μL of the methanolic fraction was injected into a Dionex UltiMate 3000 UHPLC-MS system coupled to a Thermo Scientific Orbitrap Q Exactive Focus detector (Thermo Fisher Scientific, Waltham, MA, USA) and equipped with a C18 2.1 column × 50 mm, 1.7 μm. A method based on acetonitrile gradients as the mobile phase was used ([27], with modifications). For each run an elution gradient was employed from 95% A:5% B (eluent A: 18 M water/0.1% formic acid; eluent B: 100% methanol/0.1% formic acid) to 5% A:95% B during 12 min. Positive ionization mode data (ESI+) were recorded in full-scan mode for a mass range between 200–1500 *m*/*z*. The results expressed in an *m*/*z* ratio corresponding to the surfactin family were used to identify the isoforms. Chromatogram cleaning and data treatment were performed using Excalibur 3.2 software. Chromatograms and mass spectra are expressed as relative abundance and relative percentage against the surfactin standard (Sigma Aldrich, S3523, St. Louis, MO, USA).

### 2.5. Tomato Plant Protection (ISR) against Botrytis Cinerea by Inoculation with Suspension of Bacillus velezensis

#### 2.5.1. Preparation Procedures of the Plants

In this experiment, tomato plants of the hybrid cultivar CalAce (PetoSeed Co. Chile Ltda, Santiago, Chile), 30 days old (second true leaf), were used. Seeds were germinated and grown in Speedling trays until reaching 30 days of age. Then, the roots were completely cleaned of substrate, and the plants were acclimated in a sterile Hoagland nutrient solution for three days. Next, the roots were sterilized, immersed in 70% ethanol for 1 min and then immersed in 5% sodium hypochlorite for 5 min. Finally, the roots were washed 5 times with sterile distilled water and placed in a new nutrient solution. After 5 days of recovery, plants were used in the different experiments.

#### 2.5.2. Experimental Design

Protection (ISR) experiments of tomato plants against disease caused by *B. cinerea* by inoculation of root or leaf surfaces with bacterial suspensions of the antagonist strains and spore suspension of the pathogen. Disease severity indices (DSI) caused by *B. cinerea* were evaluated to express the protection (ISR) provided by the bacterial strains. For each surface, four treatments were established: strain BBC023, strain BBC047, surfactin standard at 5 µg/mL and at 25 µg/mL (Table 1). The treatments were applied physically separate from the fungal infection (e.g., separate leaves or root/leaf), as schematically shown in Supplementary Figure S1.

Finally, a control treatment was established in which only *B. cinerea* was infected. 

For foliar application, one leaf per plant was submerged for 30 min in a bacterial suspension of ~10^7^ CFU/mL or in a surfactin solution (5 or 25 μg/mL), depending on the respective treatment. Root application was carried out by immersing the roots for 2 h in a bacterial suspension of ~10^7^ CFU/mL or in a surfactin solution (5 or 25 μg/mL).

After the establishment of the treatments, the plants were kept in a hydroponic system with a Hoagland nutrient solution and using a photoperiod of 12 h light and 12 h dark for 72 h. Afterwards, infection with *B. cinerea* was carried out according to Mariutto et al. [30] with some modifications. Small wounds were made with a dissecting needle at 6 points on a leaf (a different leaf than for the foliar treatment application, see Figure S1); at each point, 10 μL of a *B. cinerea* conidia suspension (10^5^ conidia/µL) was placed. Seventy-two hours post-infection, the disease severity around the point of injury was evaluated. A diagrammatic severity scale was used: level 1: 0–10 mm^2^; level 2: 10–20 mm^2^; level 3: 20–30 mm^2^; and level 4: 30–40 mm^2^. The progression of the disease was calculated using ImageJ software. Finally, the disease severity index was calculated using the following formula: DSI (%) = (Σ (f ∗ v)/N ∗ X) ∗ 100, where f = the number of infection points where *B. cinerea* spread beyond the injury point, v = the value on the severity scale, N = the total number of evaluated inoculation points (6 points/plant) and X = the highest value on the scale. For each treatment, 5 plants per treatment were evaluated.

### 2.6. Colonization Experiment

#### 2.6.1. Colonization in Leaves

Plants were prepared as described in Section 2.5.1 and transplanted to 500 mL pots with a mixture of peat and sterilized soil at a ratio of 1:1. After 5 days, plants were inoculated by immersing the leaves for 30 min in bacterial broth with a 10^7^ CFU/mL suspension of the BBC023 or BBC047 strain. The experimental design considered 5 plants for each strain and 10 control plants. Control leaves were immersed in MOLP medium. Colonization was evaluated at 7, 14 and 21 days post-inoculation (DPI).

#### 2.6.2. Colonization in Roots

Plants were prepared as described in Section 2.5.1 and transplanted into a rhizobox system consisting of 2 glass plates (40 cm high and 30 cm wide) with a 0.5 cm layer of substrate between the glasses. As substrate, a 1:1 mixture of peat and soil was used. The roots of the plants were submerged for 2 h in a bacterial suspension, and later, 2 plants were placed inside each rhizobox, which were then sealed. For each treatment, 12 rhizoboxes were assembled.

Colonization was evaluated at 7, 14 and 21 days post-inoculation (DPI). The evaluation was carried out at three root sections according to their growth: upper section (the third closest to the neck of the plant), middle section and lower section (last third of the roots) (Supplementary Figure S2). In each evaluation, three rhizoboxes per treatment (*n* = 3) were analyzed, of which one plant was used to obtain the population density and another was used to perform the observation with SEM. For the SEM analysis, the roots were separated according to the section. Finally, to identify the zone with the highest microbial density, each root of each section was divided into two observation points, the root tip and the middle zone, to identify the point with the highest concentration of microbial colonies.

### 2.7. Evaluation of Population Dynamics in the Rhizoplane and Phylloplane

#### 2.7.1. Colonization Density

One gram of roots or leaves was ground in a mortar with 5 mL of phosphate-buffered saline (PBS) and placed in 15 mL tubes (Corning Inc., Corning, NY, USA) with 10 mL of 1× PBS. The samples were heated for 30 min at 80 °C. From the obtained solution, a serial dilution was realized and, subsequently, 100 μL of each dilution was seeded in LB plates (triplicates) and incubated for 24 h at 37 °C. Subsequently, the CFU were counted with morphological characteristics corresponding to the strains BBC023 and BBC047. Each plant was considered as a replicate.

#### 2.7.2. Scanning Electron Microscopy (SEM)

Structural analysis of each biofilm generated by the bacterial treatments was performed using SEM at 7, 14, and 21 DPI. The root or leaf samples were fixed in 0.1 mM of sodium cacodylate (pH 7.2) and 2.5 mM of glutaraldehyde for 12 to 24 h at room temperature; they were subsequently dehydrated in a series of 50% to 100% ethanol solutions and dried with a point dryer (Critical Bal-Tec CPD 030). The dried samples were coated with a thin layer of gold prior to observation using a Leica EM SCD050 coater. For visualization, a JEOL JSM-6490 LV SEM (JEOL Ltda, Tokyo, Japan) was used. The root analysis included primary and secondary roots as well as root hairs located in each of the previously described sections (upper, middle and lower), with 5 mm root pieces being analyzed.

### 2.8. Statistical Analysis

The obtained data were analyzed by one-way ANOVA, and the means were compared using the Tukey test. All analyses were performed using the agricolae package in R [31], considering *p* < 0.05 to indicate significance.

## 3. Results

### 3.1. Attributes Associated with Colonization: Surfactin Production and Profiles, Biofilms, Motility and Adherence

**Figure 1:**
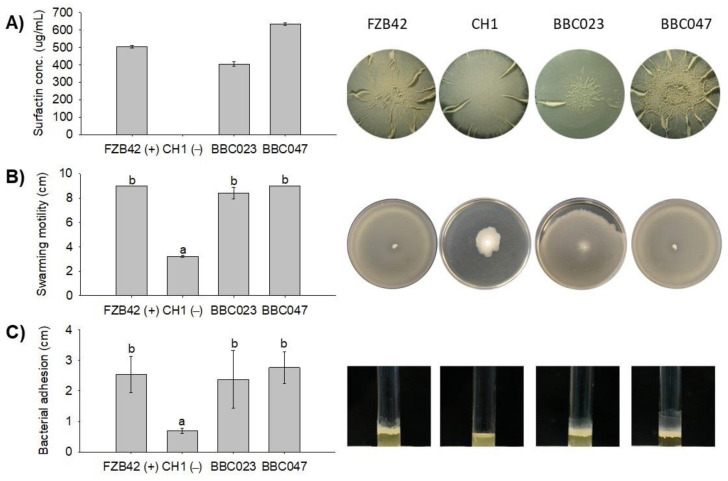
Surfactin production of *B. velezensis* strains BBC023 and BBC047, assemblage of biofilms in MSgg, motility and adherence. (**A**) Production of surfactins and biofilms in MSgg medium. (**B**) Swarming motility in a 9 cm plate with semisolid LB medium. (**C**) Surface adhesion in test tube (15 × 1 cm). Letters a and b indicate All evaluations were carried out at 48 h (images of biofilm structures were modified from Salvatierra-Martinez et al. [22]).

Surfactin production was quantified in strains BBC023, BBC047 and FZB42 (positive control) and in the mutant CH1 (negative control) (Figure 1A). Our results show that strain BBC047 produced the highest levels of surfactin (633 ± 14.1 µg/mL), followed by strains FZB42 (505 ± 0.1 µg/mL), BBC023 (405.5 ± 25 µg/mL) and CH1 (0 ± 0 µg/mL). BBC047 was also the strain with the highest capacity to assemble complex biofilms in the MSgg medium, while the CH1 strain, which does not produce surfactins, did not form secondary structures in the biofilm. BBC023 and FZB42 presented biofilms with evident secondary structures but with less complexity than that of BBC047.

Finally, strains BBC047, BBC023 and FZB42 showed no significant differences regarding their motility and adherence, while strain CH1 exhibited a significant reduction in these traits (Figure 1B,C).

**Figure 2:**
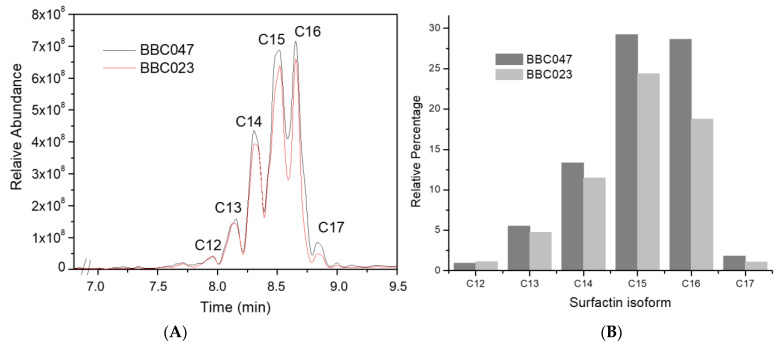
Surfactin profiles of *B. velezensis* strains BBC023 and BBC047 from UHPLC-MS. (**A**) Comparison of surfactin profiles from BBC023 and BBC047. (**B**) Relative proportion of surfactin isoforms produced by BBC023 and BBC047 compared against the surfactin standard. Detection was performed at 48 h liquid culture growth.

The comparison of the surfactin profiles revealed that both strains produce a similar pattern of isoforms (Figure 2A), ranging from C12 to C17. The relative abundance was highest for surfactin isoforms C14, C15 and C16 (>88% of the total surfactin). Compared against the surfactin standard, BBC023 produced relatively less of each isoform (except C12) than BBC047 (Figure 2B). A particularly large difference was detected for surfactin isoforms C15 and C16 with 2.5% and 8.0% less in BBC023, respectively.

Interestingly, when looking at the relative distribution of the isoforms within the profile of each strain (Figure S3), BBC023 produced slightly more of all isoforms than BBC047, except C16 and C17. The isoform distribution for strain FZB42 (Supplementary Figure S3), used as reference, displayed a shift in the relative abundance: C13 to C15 isoforms represented 87% of total surfactin, whereas C17 was not detected.

### 3.2. Protection (ISR) Experiment of Tomato by Inoculation of Plant Surfaces with Strains BBC023 and BBC047 against Disease Caused by B. cinerea

The protection (ISR) responses of tomato plants, triggered by root and leaf treatments with strains BBC023 and BBC047, were evaluated with a disease severity index of the necrotrophic fungus *B. cinerea*. These results were compared with the direct effect of different concentrations of a surfactin standard.

**Figure 3:**
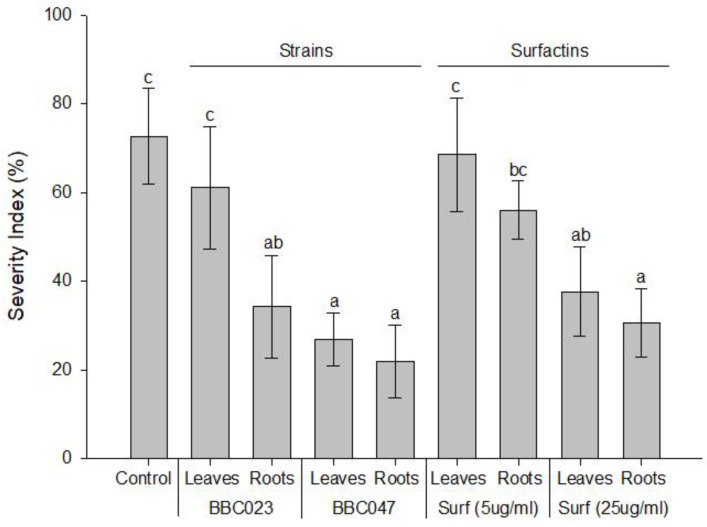
Disease severity index (DSI) (%) after *B. cinerea* infection in different bacterial and surfactin standard treatments on tomato leaves and roots via ISR (direct antagonism between treatments and pathogen was excluded). Each bar represents the mean ± standard deviation of the control index obtained from 30 observations in 5 plants. Different letters above the bars represent significant differences between the treatments according to the Tukey test (*p* < 0.05).

All root treatments (both strains and both surfactin concentrations) reduced the disease severity of *B. cinerea* with respect to the control, where the most effective treatments were BBC047, surfactin at 25 μg/mL and BBC023 (Figure 3). In leaf applications, only strain BBC047 and surfactin at 25 μg/mL reduced DSI, whereas strain BBC023 and surfactin at 5 μg/mL presented no differences from the control. Particularly, the results for strain BBC023 are noticeable, as it was not capable of activating the induction of resistance via leaf application, but when applied to the roots, it managed to reduce the severity of *B. cinerea*.

### 3.3. Colonization and Population Dynamics in the Tomato Rhizoplane and Phylloplane

The density and population dynamics of strains BBC023 and BBC047, on both the rhizoplane and phylloplane, were determined by SEM and quantified by CFU at 7, 14 and 21 DPI. We evaluated the effect of the application surface (root or leaf) on the density and population dynamics of each strain.

**Figure 4:**
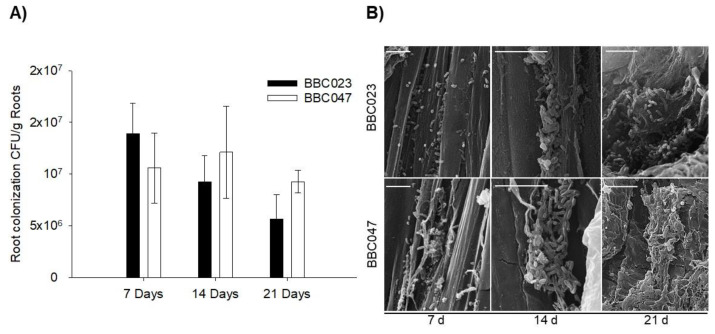
Population dynamics and colonization on the tomato rhizoplane by strains BBC023 and BBC047. (**A**) Quantification of CFU on tomato roots inoculated with BBC023 and BBC047. The bars represent the average of replicates with their standard deviation. (**B**) SEM images of root colonization. Photographs correspond to the meristematic zone of the secondary roots obtained from the lower third. Timeline represents the colony establishment (7 d), formation of extracellular matrix (14 d) and colony covered by this matrix (21 d). Scale bar represents 10 µm.

Root colonization by BBC023 and BBC047 resulted in similar population densities (Figure 4). Both strains showed a preference for the lower root section over the middle or upper section, whereas in the control treatment no colonization was observed (Supplementary Figure S4). The middle and upper sections presented only small bacterial groups up to 21 DPI (Supplementary Figures S5 and S6). On the other hand, from 7 DPI, strains BBC023 and BBC047 mainly colonized the secondary root tips of the lower section, while no marked colonization in the root hairs was registered. Finally, strain BBC047 stands out for developing marked colonization in the root maturation area.

**Figure 5:**
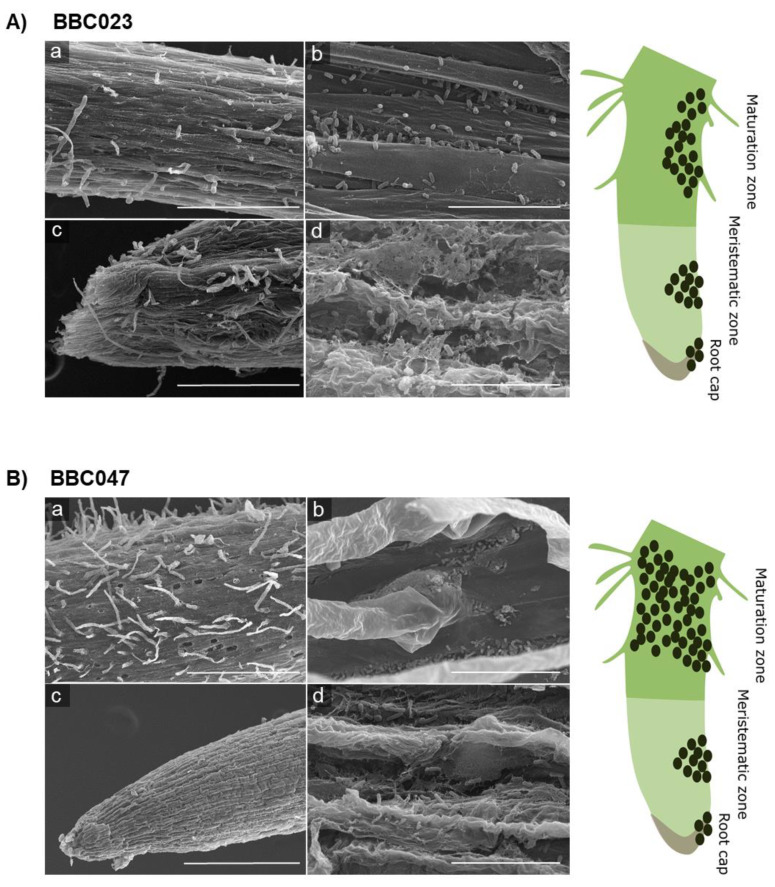
Colonization of tomato roots by *B. velezensis* strains BBC023 and BBC047 at 21 DPI. (**A**) BBC023: (**a**) secondary root maturation zone (bar 300 μm), (**b**) increased maturation zone (bar 30 μm), (**c**) zone of elongation and calyptra (bar 300 μm) and (**d**) close-up of elongation zone and calyptra (bar 10 μm). (**B**) BBC047: (**a**) secondary root maturation zone (bar 300 μm), (**b**) increased maturation zone (bar 30 μm), (**c**) elongation zone and calyptra (bar 300 μm) and (**d**) close-up of elongation zone and calyptra (bar 10 μm).

At 21 DPI, robust biofilms were observed at root tips, in the elongation zone and in the meristematic zone (Figure 5). In addition, a three-dimensional structure was observed at the meristematic and caliptral zones (Figure 5(Ac,Bc)), also highlighting the sporadic formation of microcolonies in concave zones of the primary roots. Specifically, the biofilm of BBC047 presented robust coverage, which ranged from the maturation zone to the meristematic zone of the roots. Additionally, for BBC023, numerous populations were observed in the meristematic zone, forming robust biofilms. In contrast, the close-up view of the maturation zone showed sporadic colonization but without the formation of robust films.

**Figure 6:**
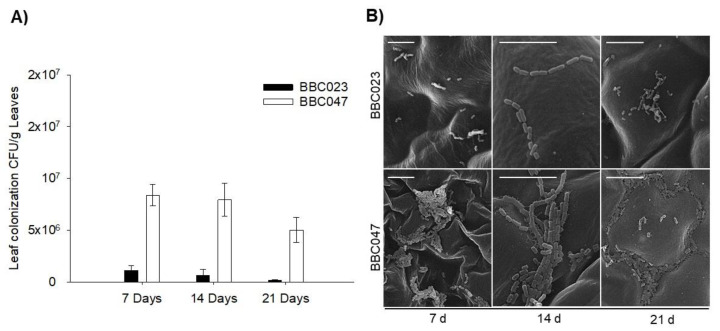
Population dynamics and colonization on the tomato rhizoplane by *B. velezensis* strains BBC023 and BBC047. (**A**) Quantification of CFU on tomato leaves inoculated with BBC023 and BBC047. The bars represent the average of replicates with their standard deviation. (**B**) SEM images of leave colonization. Scale bar represents 10 µm.

On the leaf, the strains showed opposite results. BBC023 concentration decreased after 7 days (1 × 10^7^ to 1.13 × 10^6^), with a minimum quantification of 6.67 × 10^5^ and 1.50 × 10^5^ at 14 and 21 DPI, respectively. In contrast, BBC047 presented a stable quantification, close to 107 CFU, at 7 and 14 DPI (8.38 × 10^6^), with a slight decrease only at 21 DPI (5.03 × 10^6^) (Figure 6A).

Our SEM results showed that BBC023 did not establish or organize persistent communities on the leaf, being observed at 21 DPI in only a few dispersed populations (Figure 6B). BBC047 formed numerous populations, and from 7 DPI it was possible to observe the production of an extracellular matrix. Later, at 21 DPI, structured populations in biofilms were observed in the junctions between the walls of the epidermal cells of the leaves, supported by a network formed by a type of extracellular fibrillar matrix (Figure 6B).

## 4. Discussion

Previously, our group published the first characterization of the lipopeptide production of strains BBC023 and BBC047, revealing their capability to generate all three families of LPs (surfactins, iturins and fengycins). In dual confrontation tests, synergistic antifungal activity of these LPs against *B. cinerea* was observed, revealing a direct control mechanism of this fungus [22]. Furthermore, both strains demonstrated the ability to inhibit *B. cinerea* disease via ISR when applied to tomato roots [22]. Both strains were isolated from the rhizosphere, so it is possible that they have strategies for efficient adaptation to this environment [32]. Julkowska et al. [33] reported that swarm movement (motility), adherence and biofilm production, three abilities important for efficient colonization, are linked to the production of surfactin. In this sense, the fact that BBC023 produces 1/3 less surfactin than BBC047 becomes relevant for the interpretation of our results. Despite the lower surfactin production, BBC023 and BBC047 demonstrated similar results for adherence and motility under in vitro conditions. This could be explained by the existence of other factors, which act in a coordinated manner to achieve swarming movement and adhesion, and which could compensate the lower surfactin production of BBC023 [29,34]. However, the biofilm of strain BBC047 presented a greater structural complexity than that of BBC023, which correlates with the respective surfactin production of these strains [18]. The surfactin profiles of both strains contain isoforms with carbon chains between C12 and C17, where longer carbon chains (C14, C15 and C16) represented more than 88% of the respective surfactin production. Compared to strain FZB42 (mainly producing C13 to C15 chains [24]), the surfactin profile of both strains used in this study is shifted towards longer C-chain isoforms. Particularly, these longer C-chains were reported to develop a stronger interaction with plant cell membranes and therefore are more likely involved in triggering ISR [20,35].

To elucidate the impact of the lower surfactin production of BBC023 with respect to its capacity to activate ISR, we performed a plant experiment, incorporating treatments with the application of exogenous surfactin (5 and 25 μg/mL), being the Surf 5 μg/mL treatment below the threshold reported to generate an effect on the plant [7].

Several authors mentioned a surfactin dose-dependent activation of ISR in the plant, which was confirmed by our results for the two treatments with different concentrations of exogenous surfactin [7,36]. All bacterial treatments on tomato roots activated ISR in defense against *B. cinera* disease on the leaves. In this context, Debois et al. [36] reported that the polysaccharides produced by tomato roots induce the bacterial production of surfactin. Such a root-exudate-mediated modulation of bacterial surfactin production could also explain the fact that strains BBC023 and BBC47 achieve a similar plant response, despite their differences in surfactin production under in vitro conditions.

Interestingly, for the bacterial treatments on tomato leaves the degree of disease inhibition correlated with the in vitro surfactin concentrations produced by each strain. In plants treated with BBC047, the disease severity of *B. cinerea* in root and leaf application of this strain is comparable—or in other words, the ISR response is similar. This contrasts with other studies, where surfactin showed low effectivity in activating ISR in dicot leaves [37,38]. Whereas BBC023 generated two different plant responses, on roots it triggered ISR similar to 25 μg/mL exogenous surfactin and BBC047; on leaves, disease severity is comparable to the control. These results suggest that in the interaction with the roots, other mechanisms could compensate for the lower production of surfactin and thus facilitate colonization, similar to, e.g., the production of exopolysaccharides [21,39]. Contrastingly, on leaves, the establishment of strain BBC023 might be insufficient to activate the formation of the extracellular matrix [40,41] as a consequence of its lower surfactin production [6].

At this point, it becomes important to consider the second known biological aspect of surfactin as a signaling molecule: its involvement in quorum sensing during biofilm formation [40]. Here, surfactin acts as a signaling molecule, activating the membrane receptor histidine kinase KinC and stimulating the formation of biofilms and, therefore, colonization [40,42,43]. We studied the colonization patterns of both strains in roots and leaves, correlating them with their surfactin production capacity.

After 21 DPI, in roots, BBC023 and BBC047 were able to maintain cell concentrations above 10^6^ CFU/mL, suggesting the existence of stable populations capable of inducing consistent effects [44,45]. Electron microscopy allowed us to visualize the structure and formation of the extracellular matrix of bacterial microcolonies in the different structures of the root [46]. In contrast to the findings of Fan et al. [47] for *B. velezensis* FZB42, strains BBC023 and BBC047 do not have a preference for junctions between primary and secondary roots, being found only sporadically in this area and not forming robust biofilms. However, they extensively and consistently colonized the lateral roots, forming biofilm structures.

The distribution of bacterial aggregates in different sections of the root was not uniform, similar to what was reported by Posada et al. [46]. The colonization pattern evidenced few cell aggregates (consisting of planktonic populations) in the upper and middle section, while in the lower section and specifically in the root tips robust biofilms were found.

In addition, differences in structure and robustness were detected between the biofilms formed in the maturation zone and those biofilms formed in the elongation or meristematic zones. Several authors have described that different root zones (maturation, meristematic or calyptra zones) can generate different types of compounds [48,49,50], with the highest exudation of compounds occurring in the meristematic zone [51]. Precisely in this area, a large colonization was found, although with less robust biofilm and a greater presence of planktonic cells [42]. Planktonic cells within subpopulations in biofilms are adapted for nutrient utilization rather than robustness and resistance [52]. Thus, the meristematic zone of the rhizosphere could participate in the attraction of bacteria through chemotaxis to later promote their multiplication and dispersal. On the other hand, the most robust biofilms were found in the calyptra [50]. These structures are rich in extracellular matrices, and their objective would be to protect the microbial population [53]. Interestingly, this area has the least availability of nutrients and presents the highest disturbance due to friction generated by root growth. Beyond the induction of biofilm formation, Chen et al. [40] relate surfactin to the relief of carbon catabolite repression (CCR) in *B. amyloliquefaciens* to enable the use of non-preferred carbon sources under nutrient limitation, as in the calyptra zone. These findings suggest a biological importance associated with surfactin, whose benefits for bacterial survival on plant surfaces require specific studies.

Leaf colonization patterns differed between the two strains. BBC047 presented enhanced extracellular matrix production, more structured populations and higher density. Biofilm formation occurred at the junctions of epidermal cells, similar to those reported by Zeriouh et al. [6] for the *B. amyloliquefaciens* UMAF6614 strain on melon leaves (*Cucumis sativus*). The opposite result was observed in strain BBC023. This strain showed a dramatic reduction in its population density at 7 DPI, registering only isolated and unstructured populations. This colonization pattern and behavior did not change during the experiment. In contrast to Luo et al. [19], the ability to produce other lipopeptides by BBC023 [20] did not compensate for the lower surfactin production, which resulted in an impediment to efficient and sustained leaf establishment. Similarly, the absence of a biofilm exposes microorganisms to an inhospitable environment subject to high temperatures, UV radiation and dehydration [6,54,55], reducing the population density. The leaf colonization pattern of BBC023 also could explain the results from the ISR experiment, where this strain was not able to stimulate resistance mechanisms via leaf application, as the low population density is probably insufficient to generate a physiologically relevant concentration of surfactin. Based on our results, we suggest that surfactin concentration contributes differentially not only to antifungal activity but also to biofilm formation and colonization.

## 5. Conclusions

Effective colonization of the plant surface is key for a biocontrol effect, both through antagonism and ISR. There is also a correlation between the surfactin production of a strain and its colonization, but not necessarily with its capacity to activate an ISR response. Our results suggest (1) the presence of environmental conditions that, depending on the plant surface (root or leaf), favor or limit colonization by a strain with less surfactin production; (2) the existence of other mechanisms that enable a strain with lower surfactin production to efficiently colonize, e.g., on roots; (3) that surfactin production is not a predictor for the colonization capacity of a bacterial strain; and (4) only efficient and sustained colonization enables the activation of ISR.

Finally, we conclude that the importance of surfactin lies in its impacts on biofilm formation and stable colonization, which finally enables its activity as an elicitor of ISR.

## Figures and Tables

**Table 1:** Treatments used in protection (ISR) experiment with tomato.

Treatment	Treatment Application		*B. cinerea* Application
	Root	Leaf 1	Leaf 2
Control			x
BBC023	x		x
BBC047	x		x
Surf (5 µg/mL)	x		x
Surf (25 µg/mL)	x		x
BBC023		x	x
BBC047		x	x
Surf (5 µg/mL)		x	x
Surf (25 µg/mL)		x	x

## Data Availability

The data presented in this study are included this article and its supplements.

## Supplementary Materials

The following are available online at https://www.mdpi.com/article/10.3390/microorganisms9112251/s1, Figure S1, Experimental design for ISR experiment with BBC023, BBC047 and fungal pathogen *B. cinerea*; Figure S2, Experimental design for colonization experiment in rhizobox; Figure S3, Relative proportion of surfactin isoforms produced by BBC023, BBC047 and FZB42 relative to the total amount of surfactin produced by each strain, respectively; Figure S4, Representative rhizobox and SEM photographs of control treatment at 21 DPI; Figure S5, Root photographs of the middle section at 14 DPI; Figure S6, Root photographs of the upper section at 14 DPI.
