# Supplementary material for: The Role of Surfactin Production by Bacillus velezensis on Colonization, Biofilm Formation on Tomato Root and Leaf Surfaces and Subsequent Protection (ISR) against Botrytis cinerea
Source: Microorganisms. 2021 Oct 28;9(11):2251. doi: 10.3390/microorganisms9112251 (PMC8626045; doi:10.3390/microorganisms9112251)
Supplement: Supplementary file 1 [file microorganisms-09-02251-s001.zip › microorganisms-1392530-SI.pdf]

Supplementary Figures

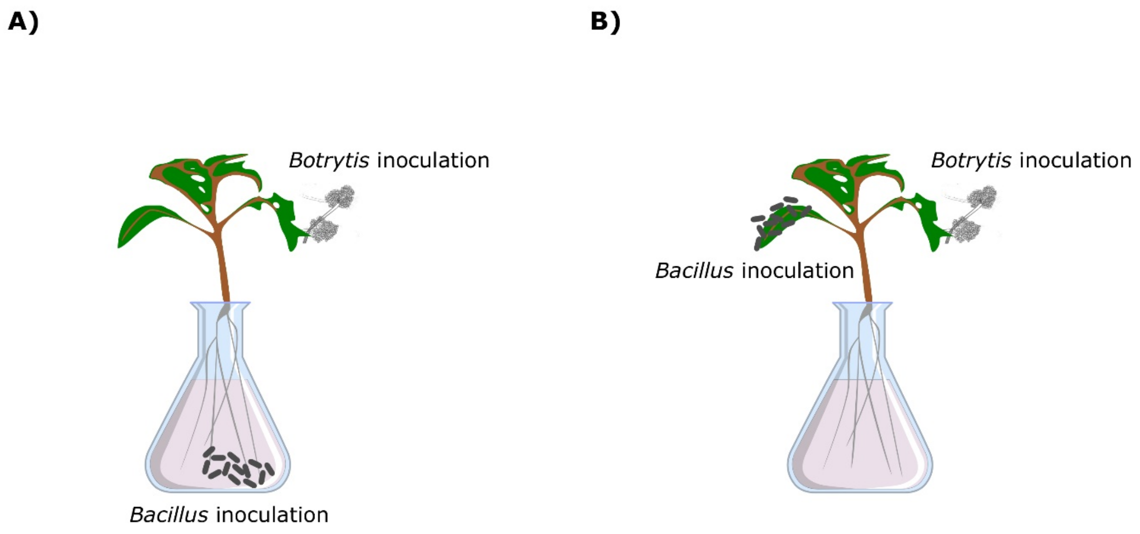

Figure S1. Experimental design for ISR experiment with BBC023, BBC047 and fungal pathogen *B. cinerea*. A) Treatment application (strains or surfactin standard) to root, fungal infection on one leaf; B) Treatment application (strains or surfactin standard) on one leaf, fungal infection on another leaf.

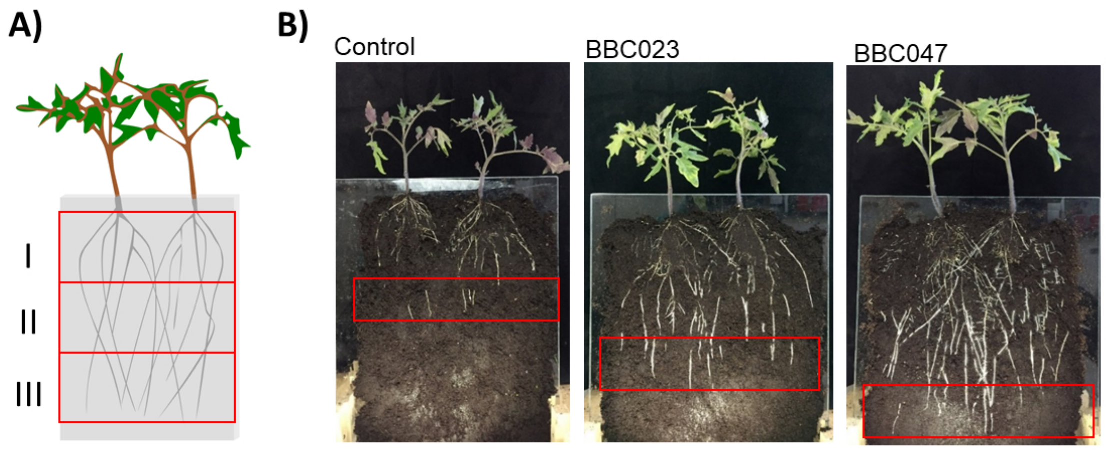

Figure S2. Experimental design for Colonization experiment in rhizobox. A) Rhizobox scheme indicating upper (I), middle (II) and lower (III) root section; B) Photographs of each treatment in rhizobox at 21 DPI indicating location of the lower root section.

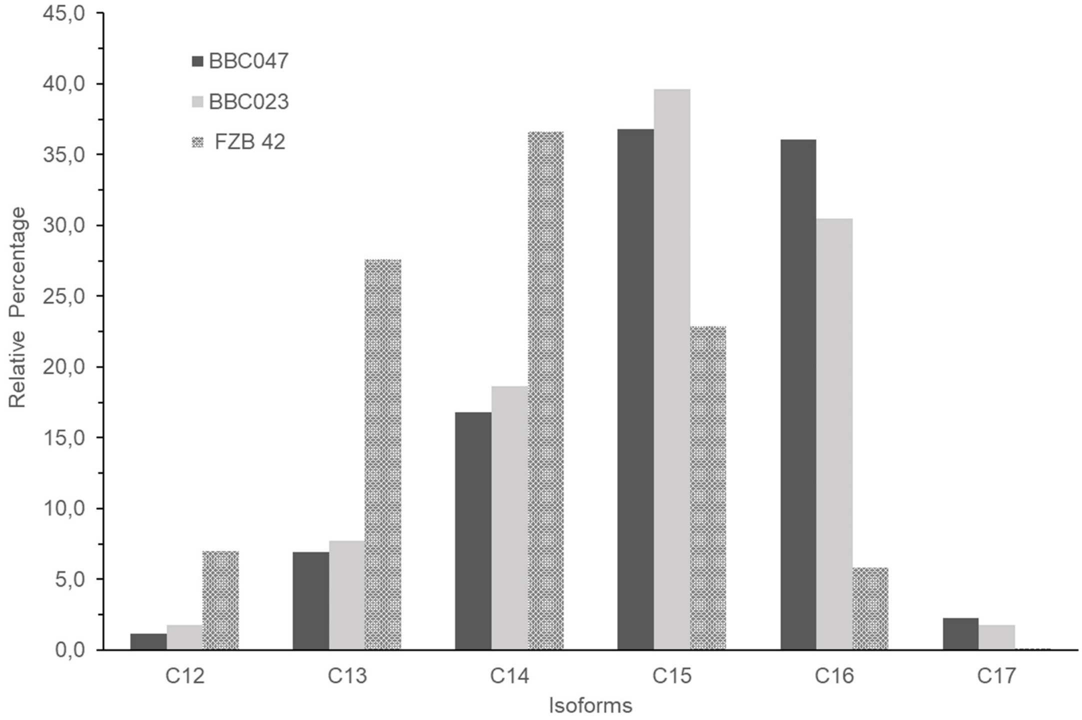

Figure S3. Relative proportion of surfactin isoforms produced by BBC023, BBC047 and FZB42 respective the total amount of surfactin produced by each strain respectively. Detection was performed at 48 h liquid culture growth.

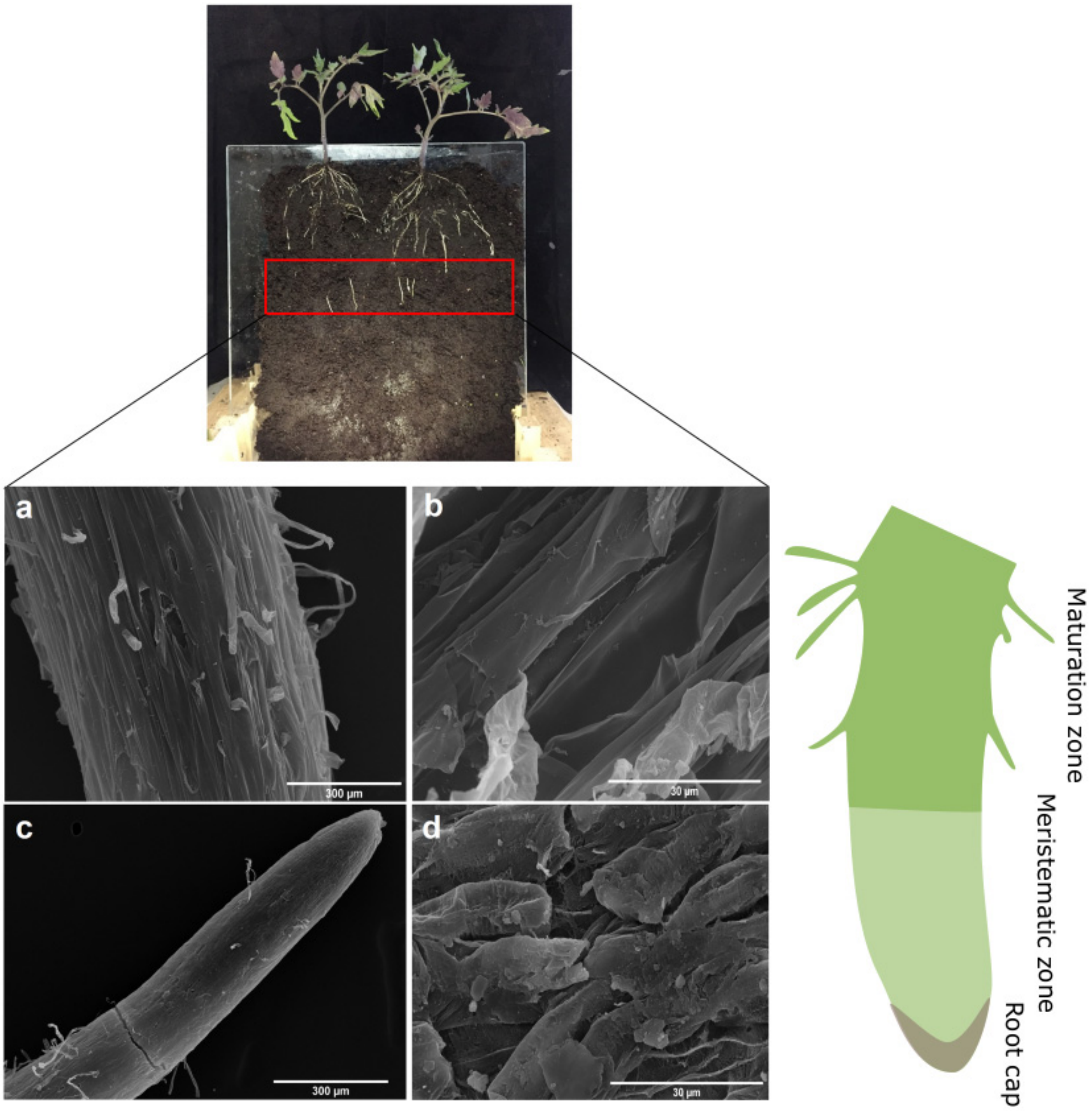

Figure S4. Representative rhizobox and SEM photographs of Control treatment at 21 DPI. a y b) secondary root maturation zone, bar 300 µm; b) Close up to the maturation zone, bar 30 µm; c) meristematic zone and calyptra, bar 300 µm; d) Close up to the meristematic zone, bar 30 µm.

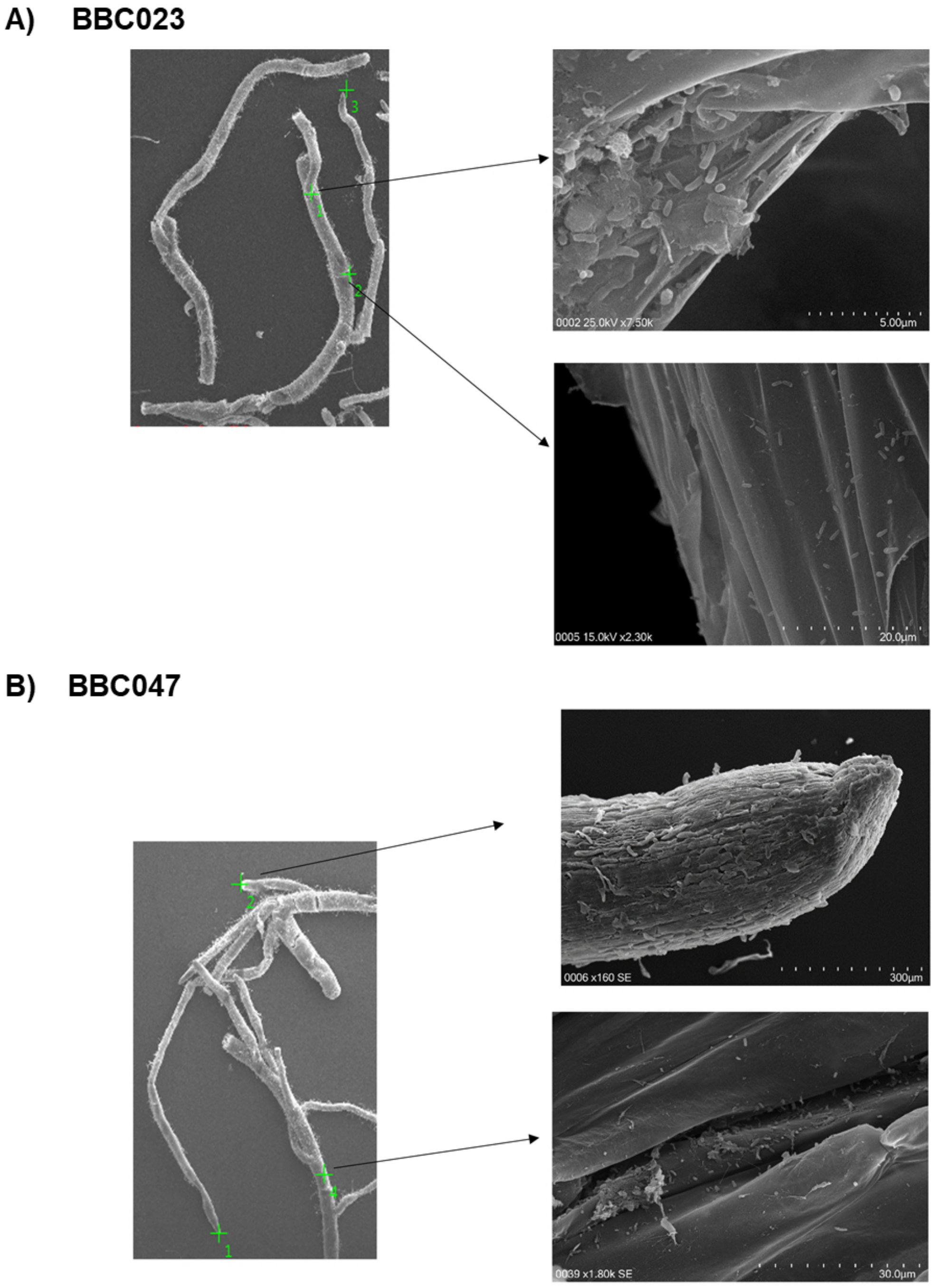

Figura S5. Root photographs of the middle section at 14 DPI. A) BBC047; B) BBC023

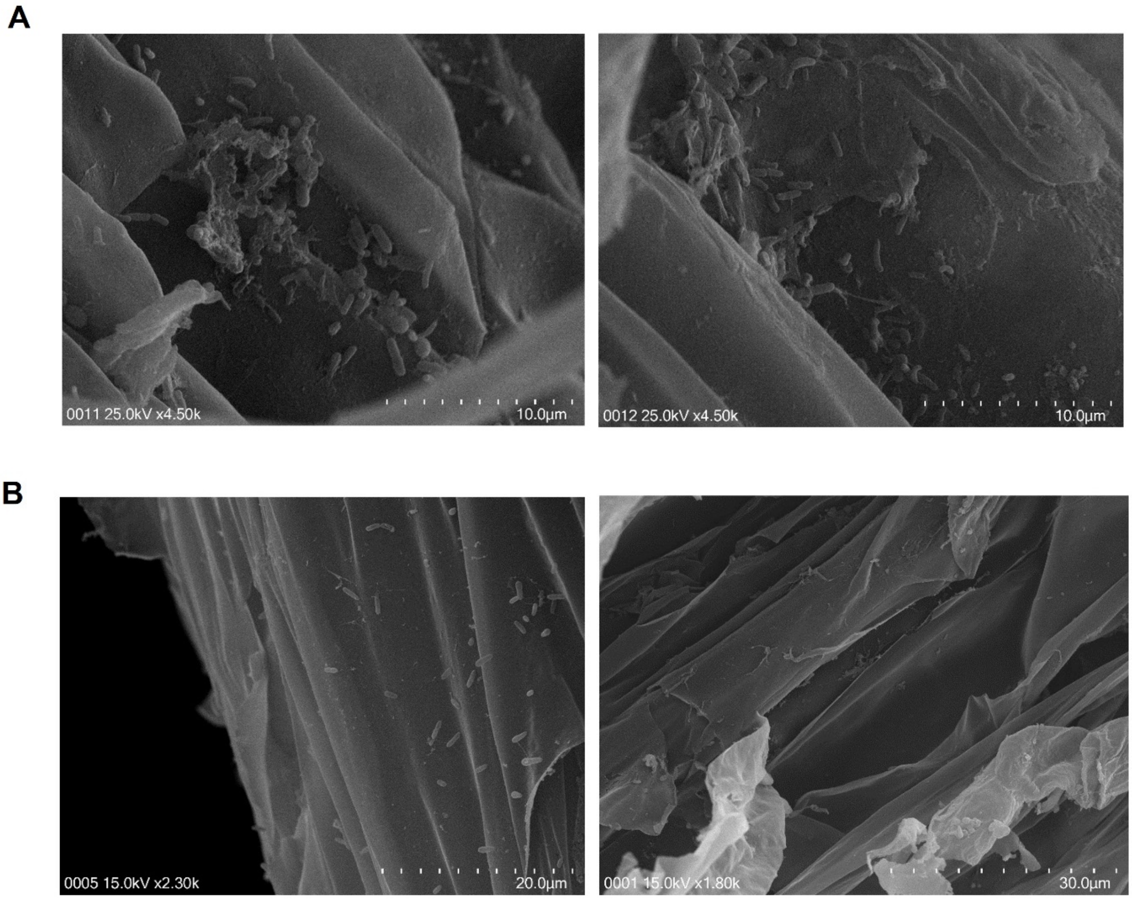

Figure S6. Root photographs of the upper section at 14 DPI. A) BBC023; B) BBC047
